# A novel way to quantify schizophrenia symptoms in clinical trials

**DOI:** 10.1111/eci.13398

**Published:** 2020-09-19

**Authors:** Oleg N. Medvedev, Michael Berk, Olivia M. Dean, Ellie Brown, Margaret H. Sandham, Joanna F. Dipnall, Robert K. McNamara, Alexander Sumich, Christian U. Krägeloh, Ajit Narayanan, Richard J. Siegert

**Affiliations:** ^1^ University of Waikato Hamilton New Zealand; ^2^ Auckland University of Technology Auckland New Zealand; ^3^ IMPACT Strategic Research Centre School of Medicine Deakin University Geelong Vic. Australia; ^4^ Emergency and Trauma Unit Department of Epidemiology and Preventive Medicine School of Public Health and Preventive Medicine Pre‐hospital Monash University Melbourne Vic. Australia; ^5^ Department of Psychiatry and Behavioral Neuroscience University of Cincinnati College of Medicine Cincinnati OH USA; ^6^ Division of Psychology Nottingham Trent University Nottingham United Kingdom

**Keywords:** clinical trial, generalizability theory, measurement, positive and negative syndrome scale, schizophrenia

## Abstract

**Background:**

A major problem in quantifying symptoms of schizophrenia is establishing a reliable distinction between enduring and dynamic aspects of psychopathology. This is critical for accurate diagnosis, monitoring and evaluating treatment effects in both clinical practice and trials.

**Materials and methods:**

We applied Generalizability Theory, a robust novel method to distinguish between dynamic and stable aspects of schizophrenia symptoms in the widely used Positive and Negative Symptom Scale (PANSS) using a longitudinal measurement design. The sample included 107 patients with chronic schizophrenia assessed using the PANSS at five time points over a 24‐week period during a multi‐site clinical trial of N‐Acetylcysteine as an add‐on to maintenance medication for the treatment of chronic schizophrenia.

**Results:**

The original PANSS and its three subscales demonstrated good reliability and generalizability of scores (G = 0.77‐0.93) across sample population and occasions making them suitable for assessment of psychosis risks and long‐lasting change following a treatment, while subscales of the five‐factor models appeared less reliable. The most enduring symptoms represented by the PANSS were poor attention, delusions, blunted affect and poor rapport. More dynamic symptoms with 40%‐50% of variance explained by patient transient state including grandiosity, preoccupation, somatic concerns, guilt feeling and hallucinatory behaviour.

**Conclusions:**

Identified dynamic symptoms are more amendable to change and should be the primary target of interventions aiming at effectively treating schizophrenia. Separating out the dynamic symptoms would increase assay sensitivity in trials, reduce the signal to noise ratio and increase the potential to detect the effects of novel therapies in clinical trials.

## 1 INTRODUCTION

A key issue for measuring psychopathological symptoms is the extent to which the underlying construct is stable or fluctuates over time.^1^ In schizophrenia, differentiation of enduring, as compared to responsive symptoms, has considerable implications for understanding underpinning biopsychosocial mechanisms, addressing problems associated with disease heterogeneity and improving health care.^2^, ^3^, ^4^ In particular, enduring negative symptoms may define a subgroup of patients differentiated on aetiology, life‐impact of illness and poor response to medication.^3^, ^4^ Poor understanding of aetiopathogenetic mechanisms of persistent negative symptoms limits the development of novel effective interventions.^4^ Generalizability theory has been used to investigate enduring (trait) and dynamic (state; eg in response to environment or time point) symptoms in the context of mood disorders^5^ and psychological interventions (eg mindfulness)^6^ and might be applied to symptoms of schizophrenia.

Understanding differences between enduring and dynamic symptom patterns may help explain the discrepancy between observed changes in negative symptoms and the widely held belief amongst clinicians that negative symptoms are resistant to treatment.^7^ Historically, positive symptoms (ie excess or distortion of normal functions, such as hallucinations or delusions) are found more amenable to change over time, while negative symptoms (ie diminution or loss of normal functions, such as affective flattening) are generally considered more enduring.^8^, ^9^ However, Savill et al,^7^ reported a meta‐analysis that suggests negative symptoms do improve over time, across several treatment conditions, to a greater extent than previously thought. Nevertheless, another meta‐analysis of 168 randomized placebo‐control trials^10^ found that while most treatments reduced negative symptoms at follow‐up relative to placebo, not to a clinically meaningful degree (ie as rated on the Clinical Global Impression Severity Scale).^11^ Understanding which negative (and positive) symptoms are more amenable to change than others might help explain these discrepancies and would contribute to the development of scales to assess dynamic and fluctuating symptoms.

In addition to evaluate clinical meaningfulness in trials, it is essential to evaluate the extent to which the outcome measure is detecting true variability in symptoms.^10^ This involves evaluating potential sources of measurement error and requires a distinction between enduring and dynamic symptom patterns. Consequently, the psychological construct of interest must be measured over multiple occasions. However, much of the research into the reliability of measures of psychological constructs has been conducted at single time points, potentially missing important insights into the dynamic nature of psychological variables.^12^ For example, in the context of personality (ie big five), repeated measurement on multiple occasions suggests that up to 25% of the variability can be attributed to transient occasion effects, rather than differences in trait levels between persons.^13^

The Positive and Negative Syndrome Scale (PANSS) is a 30‐item clinician‐rated scale that assess the presence of positive symptoms, negative symptoms and general psychopathology in people with schizophrenia.^14^ The PANSS is widely used as an outcome measure in clinical trials and generally has good reliability and validity. However, internal consistency of positive and general subscales are ‘modest’ and test‐retest reliability ranged between 0.60 to 0.80 at subscale level.^14^, ^15^ This is of particular relevance to clinical trials where clinical measures must be sensitive to true change in symptoms over time. Identification of these patterns will better identify clusters of individuals with specific patterns of schizophrenia symptoms from the general population.^16^

The reporting of composite subscale scores on the PANSS rather than individual item scores further decreases the ability to understand variation in individual symptoms over time. For example, some treatment studies select either the negative or positive subscale to identify changes in the symptoms of interest.^17^ This could lead to construct underrepresentation as the PANSS general subscale includes several possible negative symptoms and positive symptoms.^14^ This complexity increases with different factor structures of the PANSS which have been reported, such as a five‐factor solution.^18^

Establishing the true reliability of a measure requires a distinction between stable and dynamic symptom patterns, which is only possible using repeated measures.^6^ For example, Vangeneugden et al^19^ examined data from five randomized controlled trials (RCTs) across 18 countries and found that test‐retest reliability was low and measurement error higher for patients with high scores on the PANSS negative symptoms subscale. Consequently, measurement error attenuates the ability to distinguish true change in symptoms from extraneous factors.^19^

The test‐retest reliability coefficient is commonly used to evaluate consistency in scoring on psychological measures over two occasions. Test‐retest reliability does not account for multiple sources of error that may affect the observed score on a testing occasion, such as interactions between person, item and occasion.^6^ Vispoel et al^12^ demonstrated that reliability coefficients may be overinflated by as much as 24%, as single‐occasion reliability coefficients do not account for transient error (eg occasion effects such as fatigue), whereas test‐retest coefficients do not account for specific factor error (eg idiosyncratic responding due to error caused by items unrelated to the construct of interest).

Generalizability or G Theory is a sound method for evaluating specific sources of measurement error in clinical trials.^19^ While classical test theory assumes that an observation is a combination of an individual's true score and random measurement error, G Theory uses analysis of variance (ANOVA) to calculate precise estimates for the error variance due to each important measurement facet.^20^ Facet refers to any distinct element that the researcher theorizes might influence variance and error in test scores. For example, facets may be the persons tested (P), the test items (I) and the testing occasion (O). CTT restricts analysis of reliability and error variance to a single element such as the test items (Cronbach's alpha), the occasion (test‐retest) or the rater (inter‐rater) and does not allow for simultaneous evaluation of true score estimates. Importantly, G Theory also calculates separate variance components for the interactions between facets (eg Person x Occasion). In G Theory terms, the variance associated with participants or persons is considered the central concern and is known as the differentiation facet with other facets (eg items, occasion, rater) viewed as sources of measurement error.

Applying G Theory involves two stages: G‐study and the D‐study. The G‐study involves a factorial ANOVA corrected for the type of sampling involved (ie random, fixed or mixed) and estimates a G‐coefficient reflecting generalizability of the test scores across persons and situations. Bloch and Norman (p. 968)^20^ describe the G‐coefficient as the ratio of ‘signal’ to ‘noise’ or ‘true variance’ to ‘true + error variance’. The D‐study, or Decision‐study, allows the researcher to estimate the impact on reliability of variations in different facets such as increasing the number of participants or the number of items in a scale, and hence decide on the appropriate measurement protocols.

Only a few studies have applied G Theory to investigate the psychometric properties of the PANSS, and none distinguished between dynamic and stable symptom patterns using a longitudinal (repeated measures) design. Vangeneugden et al^19^ argued that G Theory represents a powerful psychometric approach that has particular potential for clinical trials and demonstrated this in a G Theory study of the PANSS used across different countries. Khan et al^21^ applied G Theory to data from a failed clinical trial to determine the major sources of unreliability (Patient, Rater, Occasion) and concluded that the major source of variability in scores was Rater followed by Rater x Occasion. Being able to parse stable and dynamic symptoms may allow the development of a dynamic subscale which might increase assay sensitivity and increase the likelihood of detecting between‐group change in trials of novel therapies for schizophrenia. In the present study, we apply G Theory to identify stable and dynamic symptom progression within the context of a clinical trial involving a cohort of patients diagnosed with schizophrenia to evaluate the psychometric properties of the PANSS, its subscales and individual items.

## 2 METHODS

### 2.1 Participants

Participants were 107 patients with a longstanding diagnosis of schizophrenia (mean duration of illness, 12.2 ± 8.9 years) aged between 18 and 65 years, and randomized to N‐acetyl cysteine (NAC, n = 54) or placebo (n = 53) in a controlled trial that was reported by Berk et al^22^ and approved by the authors institutional ethics committee. This subsample satisfies requirements for reliability study in medical research^23^ and was selected from a larger sample of 140 patients based on the availability of complete PANSS data from five separate assessments collected at two‐week intervals (Baseline, week 2, week 4, week 6 and week 8). Exclusion of 33 participants with missing data was assumed as random because missing data were completely at random. We have also estimated if the reduced sample was statistically different from the full sample using Monte Carlo stimulation that indicated no significant difference with upper bound overlapping *P*‐value cut‐off point of .05 [99%CI; 0.04, 0.05]. Patients were required to have residual symptoms, as defined by a baseline PANSS Total score of ≥55 or at least two of the positive and/or negative items being ≥3 despite maintenance treatment with atypical antipsychotic medications. The inclusion criteria were selected so that participants had current symptoms (mildly ill +) based on the literature of trials.^23^ Both the placebo and NAC groups exhibited significant baseline‐week 8 reductions in the PANSS Positive, Negative, General and Total scores. There were no significant treatment group differences for baseline‐week 8 change in all PANSS measures (*P > *.05), and these groups were therefore combined for the present analysis.

### 2.2 Measures

The PANSS^14^ is a 30‐item rating scale with seven positive symptom items (eg delusions, hostility), seven negative symptom items (eg blunted affect, poor rapport) and 16 representing general psychopathology (eg anxiety, depression). Each item or symptom is rated on a seven‐point scale representing increasing psychopathology from 1 (absent) to 7 (extreme). Scores are obtained for positive symptoms, negative symptoms, general psychopathology and a composite score. Raters were either clinical psychologists or medical practitioners who had undergone training on the PANSS.

### 2.3 Generalizability analysis

**FIGURE 1:**
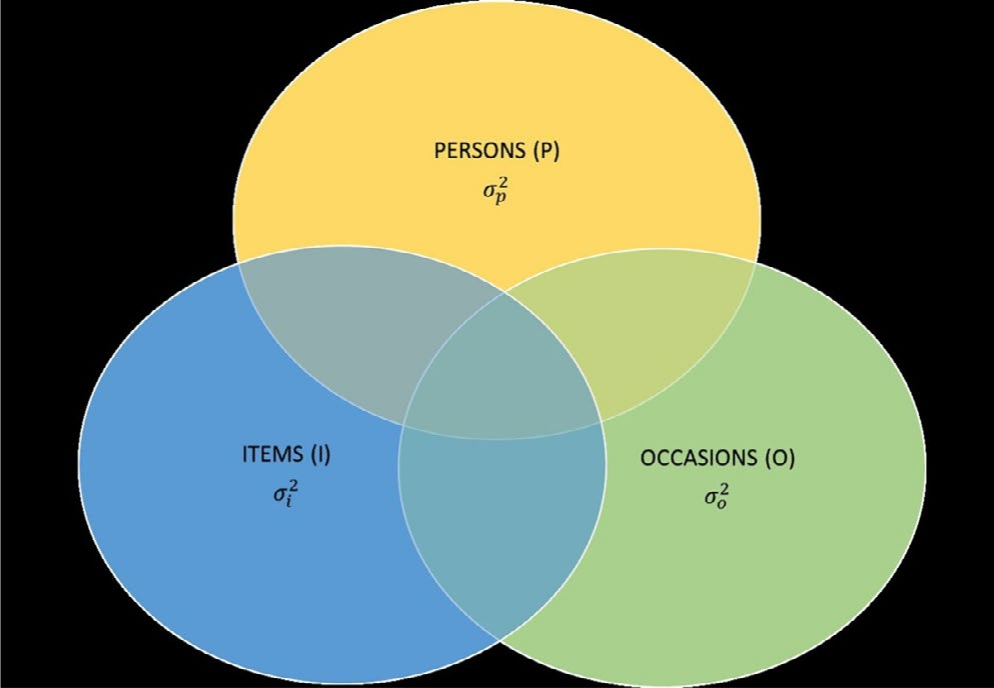
Venn Diagram of Two‐Facet Design (P x I x O)

Generalizability analyses were conducted following the guidelines described elsewhere (Gardinet et al, 2009) and employed EduG 6.1‐e software.^24^ Both the G (generalizability) and D (decision) studies used a persons (P), by item (I), by occasion (O) random effects design expressed as P × O × I, where the I facet is fixed and the P and O facets are infinite Table 1. This two‐facet design considered persons as the object of measurement, defined as the differentiation facet and not a source of error, and items and occasions as the instrumentation facets (Figure 1).^25^ Error variance due to person‐occasion interaction in a scale score can be interpreted as reflecting a dynamic component or individual state.^6^

**TABLE 1:** Components definitions for two‐facet Generalizability analysis person x item x occasion (P x I x O)

Persons (P)	Person universe score *p* (averaged deviation from grand mean over items and occasions)
Items (I)	Item effect *i* (averaged deviation from the grand mean over persons and occasions)
Occasions (O)	Occasion effect *o* (averaged deviation from grand mean over persons and items)
P x I	Effect of interaction between person *p* and item *i* averaged over occasions
P x O	Effect of interaction between person *p* and occasion *o* averaged over items
I x O	Effect of interaction between item *i* and occasion *o*
P x I x O, *e*	Effect of interaction between person *p*, item *i* and occasion *o,* containing a random error *e*

Variance components for each facet and their interactions were computed based on traditional ANOVA estimates using equations introduced by Brennan.^26^ Whimbey’s correction was applied to traditional ANOVA estimates that consider finite facets, such as items, that are not derived from infinite populations.^25^ Whimbey’s correction has no effect on random facets (eg persons) and is expressed as ((N(f)−1)/N(f)), where N(f) is the population size of the f facet in the G‐study design. The unique contribution of each facet to the total variance of universe scores was estimated using generalizability analysis and included relative and absolute error variance and G‐coefficients for the differentiation facet (persons). The relative G‐coefficient (Gr) is commonly expressed as ρ^2^, ϖ^2^ only considers variance directly related to the object of measurement. The absolute G‐coefficient (Ga) is equivalent to Phi (Φ) and accounts for other variance sources (eg item x occasion interaction) that may influence an absolute measure indirectly.^25^ To measure scale/item ability to assess dynamic and enduring symptoms, a state component index (SCI) and trait component index (TCI) were computed respectively using formulas developed by Medvedev et al^6^ to reflect the proportion of variance attributed to dynamic and enduring aspects in a measure. The D‐study investigated properties of individual items representing specific symptoms by reducing and varying facet designs aimed at optimizing measurement. Reporting of the study conforms to broad EQUATOR guidelines (Simera et al. January 2010 issue of EJCI).^27^

## 3 RESULTS

Descriptive statistics were computed for the five separate PANSS assessments with the current sample and are presented in Table 2. The total PANSS and Negative and General symptom subscales displayed good internal consistency across all five assessment points, ranging from 0.77 to 0.89. Slightly lower but acceptable values (0.70‐0.74) were observed for the Positive symptoms PANSS subscale. There was a statistically significant decrease in positive, general and overall symptoms at all assessment points compared to baseline.

**TABLE 2:** Means, standard deviation (SD), Cronbach's alpha and test‐retest coefficients for the PANSS subscales and the total scale (n = 107 × 5 occasions)

Scale/Assessment	Baseline	2 Weeks	4 Weeks	6 Weeks	8 Weeks
**PANSS Positive**					
Mean (SD)	15.76 (5.28)	14.96* (5.08)	14.47** (5.38)	14.15**(5.29)	13.78**(5.18)
Cronbach's alpha	0.70	0.70	0.73	0.74	0.72
Test‐retest *(r*)^a^	‐‐	0.87	0.79	0.78	0.79
**PANSS Negative**					
Mean (SD)	15.87 (6.13)	15.71 (6.19)	15.42 (6.17)	15.10* (6.03)	14.83** (6.10)
Cronbach's alpha	0.81	0.84	0.84	0.86	0.86
Test‐retest *(r*)^a^	‐‐	0.83	0.81	0.81	0.77
**PANSS General**					
Mean (SD)	31.86 (8.23)	30.05**(8.30)	28.93** (8.00)	28.56**(8.69)	28.08**(8.30)
Cronbach's alpha	0.77	0.8	0.79	0.82	0.81
Test‐retest *(r*)^a^	‐‐	0.84	0.76	0.79	0.75
**PANSS Total**					
Mean (SD)	63.49 (15.60)	60.72**(15.95)	58.82** (15.87)	57.81**(16.30)	56.69**(16.03)
Cronbach's alpha	0.86	0.88	0.88	0.89	0.89
Test‐retest *(r*)^a^	‐‐	0.86	0.82	0.81	0.78

Note

Mean difference is significant compared to the baseline.

^a^ Test‐retest bivariate correlations between assessment and the baseline.

* *P* < .05.

** *P* < .01.

### 3.1 G‐Study

Results of the G‐Study analysis of the PANSS total scale, 3‐ and 5‐factor models subscales are presented in Table 3. The total PANSS scale demonstrated acceptable generalizability of scores across occasions and sample population (Gr = 0.93/Ga = 0.77), with a low proportion of variance attributed to dynamic fluctuations (SCI = 0.13). The PANSS Positive, Negative and General Symptoms subscales showed slightly lower but still acceptable generalizability of scores across persons and occasions (Gr = 0.77‐0.84; Ga = 0.72/0.74). All subscales of the 5‐factor model appeared less reliable with both Gr and Ga below 0.70 bench mark.

**TABLE 3:** G‐study estimates of variance components^1^

Scale/Subscale	P	I	O	PxI	PxO	IxO	PxIxO	G‐r	G‐a	SCI
PANSS Total	0.07	<0.00	0.02	<0.00	0.01	<0.00	<0.00	0.93	0.77	0.13
Positive Symptoms	0.18	<0.00	0.01	0.02	<0.00	<0.00	0.02	0.80	0.74	<0.00
Negative Symptoms	0.17	<0.00	0.01	0.03	<0.00	<0.00	0.02	0.77	0.72	<0.00
General Symptoms	0.09	<0.00	0.01	0.01	0.01	<0.00	0.01	0.84	0.74	0.10
**5‐Factor Model**										
Positive Symptoms	0.10	<0.00	0.02	0.03	0.01	0.01	0.03	0.65	0.57	0.09
Negative Symptoms	0.12	<0.00	0.01	0.04	<0.00	0.01	0.03	0.65	0.60	<0.00
Disorganization	0.08	<0.00	0.02	0.02	<0.00	<0.00	0.02	0.66	0.57	<0.00
Excitement	0.02	<0.00	0.02	0.07	0.01	<0.00	0.00	0.15	0.12	0.33
Emotional Distress	0.10	<0.00	0.00	0.04	<0.00	0.01	0.04	0.57	0.52	<0.00

^1^ Differentiation variance component of person (P); Absolute error variance of item (I), occasion (O), interaction between person and item (PxI), person and occasion (PxO), item and occasion (IxO) and person, item and occasion (PxIxO); Relative G‐coefficient (Gr); Absolute G‐coefficient (Ga); and State Component Index (SCI) for P x O x I design including the PANSS total, 3SCI= (n = 107).

### 3.2 D‐Study

The D‐study aimed to identify the PANAS items that are sensitive to dynamic changes. Table 4 includes variance components of person (enduring symptoms), person x occasion interaction (dynamic symptoms), and SCI values to determine items reflecting dynamic symptoms. SCI values ranged from 0.27 to 0.50 with a mean of 0.38 indicating that the majority of variance in the symptoms is explained by enduring patterns. There are five items reflecting the most stable symptoms with SCI ≤0.30 including poor attention, blunted affect, delusions, poor rapport, and mannerisms and posturing. However, there are also nine items reflecting the most dynamic symptoms with larger proportion of state‐related variance (SCI = 40‐50) including: hallucinatory behaviour, guilt feeling, somatic concern, preoccupation, grandiosity, disturbance of volition, motor retardation, uncooperativeness and lack of judgement and insight. Attempts to increase the scale sensitivity to temporal changes by analysing various combinations of individual items that are the most sensitive to dynamic change with SCI above the mean of 0.38 were unsuccessful due to larger proportion of trait‐related (person) variance (eg SCI < 0.30).

**TABLE 4:** Variance components and the SCI for the individual PANSS items^1^

Items	Symptoms/Variance	P	PO	SCI^c^
G11	Poor attention	0.61	0.22	0.27
N1	Blunted affect	0.45	0.18	0.29
P1	Delusions	0.53	0.21	0.29
N3	Poor rapport	0.39	0.16	0.29
G5	Mannerisms and posturing	0.53	0.23	0.30
N6	Lack of spontaneity and flow	0.52	0.23	0.31
G16	Active social avoidance	0.33	0.16	0.33
N7	Stereotyped thinking	0.40	0.20	0.33
P4	Excitement	0.37	0.19	0.34
N5	Difficulty in abstract thinking	0.33	0.17	0.34
P2	Conceptual disorganization	0.35	0.19	0.35
P7	Hostility	0.43	0.24	0.36
G10	Disorientation	0.29	0.17	0.37
G9	Unusual thought content	0.22	0.13	0.37
N4	Apathetic social withdrawal	0.25	0.15	0.38
P6	Suspiciousness/persecution	0.27	0.17	0.39
N2	Emotional withdrawal	0.38	0.24	0.39
G4	Tension	0.38	0.24	0.39
G6	Depression	0.38	0.24	0.39
G2	Anxiety	0.30	0.19	0.39
G14	Poor impulse control	0.28	0.18	0.39
G12	Lack judgement and insight	0.32	0.21	0.40
G8	Uncooperativeness	0.27	0.18	0.40
G7	Motor retardation	0.30	0.23	0.43
G13	Disturbance of volition	0.33	0.26	0.44
P5	Grandiosity	0.16	0.13	0.45
G15	Preoccupation	0.17	0.14	0.45
G1	Somatic concern	0.27	0.24	0.47
G3	Guilt feeling	0.16	0.15	0.48
P3	Hallucinatory behaviour	0.20	0.20	0.50

^1^ Differentiation variance of person (P); Absolute error variance of person and occasion interaction (P x O); and the State Component Index (SCI).

## 4 DISCUSSION

The present study applied G Theory to evaluate reliability and quantify differences between dynamic and enduring symptom patterns in the PANSS within the context of a clinical trial involving a cohort of patients diagnosed with schizophrenia. Overall, the PANSS total scale demonstrated acceptable reliability and was superior in performance when compared to the individual subscales. The total PANSS score reflected enduring symptoms to a greater, and dynamic symptoms to a lesser, extent and was not affected by any other measurement error. The three individual PANSS subscales Positive Symptoms, Negative Symptoms and General Symptoms showed acceptable reliability and generalizability of scores across occasions and population of the sample. However, evaluation of the five‐factor structure of the PANSS^18^ revealed that all subscales had low reliability below widely the accepted 0.70 benchmark meaning that measurement error exceeded 30% for these subscales^20^ and consequently the use of a five‐factor solution is not supported.

The current study distinguished between stable and more dynamic symptoms of schizophrenia using G Theory. The symptoms operationalized by the PANSS were overall more enduring, and the most stable symptoms included symptoms located on the general scale such as poor attention and mannerisms and posturing; symptoms on the positive scale of delusions; and symptoms on the negative scale of blunted affect and poor rapport. However, the most dynamic symptoms on the positive scale were identified such as hallucinatory behaviour and grandiosity as well as symptoms on the general scale including guilt feeling, somatic concern, preoccupation, disturbance of volition, motor retardation, uncooperativeness and lack of judgement and insight.

It is notable that both the placebo and NAC groups exhibited significant baseline‐week 8 reductions in PANSS Total, Positive, General scores, but neither group exhibited significant reductions in PANSS Negative scores. While negative symptoms were generally stable, if symptoms at the subscale level were solely reported, then important dynamic changes occurring would be overlooked. For example, some symptoms on the negative subscale were dynamic, as were negative symptoms located on the general subscale (Motor retardation and Disturbance of volition). While the PANSS and its subscale scores can be used to evaluate the overall symptomatic change, individual item scores may be more useful than the scale and subscales scores to monitor more dynamic or enduring symptoms as evaluated in Table 3. Examining individual symptom patterns as opposed to subscale and total scores enables valuable detailed evaluation of symptom patterns and response to treatment, yet has not been widely reported in schizophrenia research.

Although G Theory has previously been applied to the PANSS,^20^ no studies to date have focused on distinguishing between dynamic and enduring symptoms using an extensive longitudinal measurement design over five time points. Establishment of which symptoms are most stable and which are relatively dynamic needs further exploration. While blunted affect and poor rapport were observed to be stable in the present study, Fusar‐Poli et al^10^ observed global changes in negative symptoms over time. However, they used the traditional CTT methodology that is unable to separate clearly between variance due to change at the group level from variability of individual items reflecting symptoms at individual level while controlling for person variance.^5^ Therefore, Fusar‐Poli et al^10^ findings of dynamic negative symptoms may illustrate the ability of negative items to reflect change at group level, but a poorer ability to reflect individual change accounting for unwanted error variance. In the present study using more robust G Theory method, both stable and dynamic symptoms were found on the positive and general subscales, which is not consistent with prior research based on CTT method and indicating that positive symptoms are overall more dynamic.^9^ This inconsistency is likely attributed to limitations of CTT and highlights the needs for further replication these findings using G Theory with different sample varying of symptoms severity. Furthermore, it highlights the importance of evaluating individual item level change in symptoms rather than scale and subscales scores.

There are a number of potential limitations to the present study. Although combining placebo and treatment groups may confound interpretation of stable vs dynamic features in the context of treatment, the inclusion of a variety of symptom levels was advantageous. Of note, baseline to week eight change for all PANSS measures did not differ significantly between groups, and the present analysis does identify stable vs dynamic symptom domains within the context of a clinical trial. Second, patients in this study were treated with an atypical antipsychotic medication, which may differentially modify PANSS symptoms. N‐acetyl cysteine is particularly likely to affect glutamate receptors and may be expected to have a greater effect on reality distortion and thought disorder symptoms. Therefore, assessment of medication‐naive subjects, either at the initial onset of psychosis or subjects at ultra‐high risk for developing schizophrenia, would represent an important extension of G theory and enable further exploration of stable and dynamic symptoms longitudinally. We also excluded 33 participants with missing data from the study sample that could potentially represent a selection bias. However, missing data were completely at random suggesting random selection of participants for the study was assumed and there was no evidence that the reduced sample was statistically different from the full sample.

The results of this study inform future studies to develop an instrument for distinct and reliable measurement of dynamic and enduring schizophrenia symptoms. The current investigation of the PANSS provides a methodological pathway for the development of such instruments that would enhance clinical care by permitting accurate monitoring of patients symptoms over time using two mechanisms: first, by applying a dynamic symptoms scale to evaluate individual symptom change, and second, by assessing the overall psychopathology level by simultaneously applying an enduring symptoms measure. This is particularly useful with adolescent populations for differential diagnosis between schizophrenia and the paranoid and dissociative symptoms associated with borderline personality disorder,^28^ and to identify those at clinical high risk to develop psychosis.^29^

## 5 CONCLUSION

The present study highlights the value of G Theory for quantifying stable and dynamic symptoms using the PANSS within the context of a clinical trial. The majority of symptoms represented by the PANSS were stable with the most enduring being poor attention, delusions, blunted affect and poor rapport. However, there are also more dynamic symptoms with 40%‐50% of variance explained by the patients’ transient state including grandiosity, preoccupation, somatic concerns, guilt feeling and hallucinatory behaviour. These dynamic symptoms are more amendable, which makes them the primary target of interventions aiming to effectively treat schizophrenia.

The PANSS and its three subscales demonstrated acceptable reliability and generalizability in measuring stable and dynamic symptoms of schizophrenia. The use of subscales within the 5‐factors model of PANSS was not supported in this study and should be treated cautiously in other clinical trial contexts. Additional G Theory analyses of the PANSS are warranted using prodromal and medication‐naive patients in a naturalistic setting to identify stable vs dynamic features associated with the progression of schizophrenia.

## Data Availability

Additional data can be requested from the first author by email: oleg.medvedev@waikato.ac.nz
